# The prevalence of substance use disorders and substance use in anorexia nervosa: a systematic review and meta-analysis

**DOI:** 10.1186/s40337-021-00516-3

**Published:** 2021-12-11

**Authors:** Daniel J. Devoe, Gina Dimitropoulos, Alida Anderson, Anees Bahji, Jordyn Flanagan, Andrea Soumbasis, Scott B. Patten, Tom Lange, Georgios Paslakis

**Affiliations:** 1 Department of Psychiatry, Cumming School of Medicine, Mathison Centre for Mental Health Research and Education, University of Calgary, 3280 Hospital Drive NW, Calgary, AB T2N 4Z6 Canada; 2 Faculty of Social Work, University of Calgary, Calgary, Canada; 3 Ruhr-University Bochum, University Clinic for Psychosomatic Medicine and Psychotherapy, Campus East-Westphalia, Lübbecke, Germany

**Keywords:** Anorexia nervosa, Substance use disorders, Substance use, Eating disorders, Comorbidity, substance misuse, drug abuse/dependence

## Abstract

**Aim:**

Individuals with anorexia nervosa (AN) often present with substance use and substance use disorders (SUDs). However, the prevalence of substance use and SUDs in AN has not been studied in-depth, especially the differences in the prevalence of SUDs between AN types [e.g., AN-R (restrictive type) and AN-BP (binge-eating/purge type]. Therefore, this systematic review and meta-analysis aimed to assess the prevalence of SUDs and substance use in AN samples.

**Method:**

Systematic database searches of the peer-reviewed literature were conducted in the following online databases: MEDLINE, PsycINFO, Embase, and CINAHL from inception to January 2021. We restricted review eligibility to peer-reviewed research studies reporting the prevalence for either SUDs or substance use in individuals with AN. Random-effects meta-analyses using Freeman–Tukey double arcsine transformations were performed on eligible studies to estimate pooled proportions and 95% confidence intervals (CIs).

**Results:**

Fifty-two studies met the inclusion criteria, including 14,695 individuals identified as having AN (mean age: 22.82 years). Random pooled estimates showed that substance use disorders had a 16% prevalence in those with AN (AN-BP = 18% vs. AN-R = 7%). Drug abuse/dependence disorders had a prevalence of 7% in AN (AN-BP = 9% vs. AN-R = 5%). In studies that looked at specific abuse/dependence disorders, there was a 10% prevalence of alcohol abuse/dependence in AN (AN-BP = 15% vs. AN-R = 3%) and a 6% prevalence of cannabis abuse/dependence (AN-BP = 4% vs. AN-R = 0%). In addition, in terms of substance use, there was a 37% prevalence for caffeine use, 29% prevalence for alcohol use, 25% for tobacco use, and 14% for cannabis use in individuals with AN.

**Conclusion:**

This is the most comprehensive meta-analysis on the comorbid prevalence of SUDs and substance use in persons with AN, with an overall pooled prevalence of 16%. Comorbid SUDs, including drugs, alcohol, and cannabis, were all more common in AN-BP compared to AN-R throughout. Therefore, clinicians should be aware of the high prevalence of SUD comorbidity and substance use in individuals with AN. Finally, clinicians should consider screening for SUDs and integrating treatments that target SUDs in individuals with AN.

**Plain English Summary:**

Individuals with anorexia nervosa (AN) may also present with substance use or have a substance use disorder (SUDs). Thus, we conducted a systematic review and meta-analysis to determine the prevalence of substance use and substance use disorders in individuals with AN. We examined published studies that reported the prevalence of either substance use or SUDs in individuals with AN. We found that substance use disorders had a 16% prevalence and that drug abuse/dependence disorders had a prevalence of 7% in those with AN. These rates were much higher in individuals with binge-eating/purging type compared to the restrictive AN. However, many specific substance use disorders and substance use types were low in individuals with AN. Nonetheless, clinicians should be aware of the high prevalence of SUD comorbidity and substance use in individuals with AN.

**Supplementary Information:**

The online version contains supplementary material available at 10.1186/s40337-021-00516-3.

## Introduction

Eating disorders (EDs) are associated with a series of comorbidities, including depression, anxiety, personality disorders, and substance use disorders (SUDs) [29]. A recently published meta-analysis on the prevalence rates examining the comorbidity of SUDs in EDs found that the pooled prevalence of SUDs in EDs was 22% [6], with the prevalence of EDs among individuals seeking treatment for SUDs being 35%. Thus, the prevalence of EDs in individuals with SUDs appears to be ten times higher than the prevalence of EDs in the general population [21], with the prevalence of SUDs among individuals with EDs in treatment between 25 and 50% [22, 57].

Research shows weaker associations between restrictive types of EDs [e.g., Anorexia Nervosa (AN)] and SUDs, although mechanisms of addiction may also be at play in AN [26, 44, 60, 61]. For example, cues such as pictures of underweight bodies or physical activities are reinforcers and are associated with activation/sensitization of brain structures of reward [24, 27], while other cues such as pictures of high-calorie foods do not go along with approach reactions [45]. Such findings have led to the “reward-centered” model, which posits that food cues are processed as aversive, but disorder-compatible signals are processed positively and activate the mesolimbic reward system [44]. Subsequently, restrictive eating behaviors and disorder-compatible behaviors in AN (e.g., fasting, physical activity, frequent weighing, etc.) acquire the character of automated habitual behaviors and may lead to maintenance of the disorder. Thus, comparable to addictive disorders, a transition from goal-directed to automatic habitual behaviors in response to disorder-compatible stimuli may be at play. In addition, innovative treatment approaches (such as repetitive transcranial magnetic stimulation and deep-brain stimulation), targeting brain activity associated with the regulation of both food and addictive substance intake, appear to be emerging and to show promising results [16, 28, 48, 49], which may help with the reduction of symptoms in both AN and SUDs.

Overall, despite evidence demonstrating similarities in the underlying mechanisms, associations, and prevalence of AN and SUDs, SUD in AN have not been studied in-depth in a systematic review and meta-analysis. In addition, information on the prevalence of substance use (at any frequency) in AN may help contextualize the specific patterns of substances that are more likely to lead to functional consequences in persons with AN. Therefore, this systematic review and meta-analysis aimed to: (1) assess the prevalence rates of comorbidities between AN and SUDs or substance use (2) assess the prevalence of SUDs and substance use by AN type (AN-R and AN-BP); and (3) assess the quality of peer-reviewed literature to date. This is necessary to understand AN comorbidities, clinical indicators, and outcomes and inform future treatment planning.

## Method

### Protocol and guidelines

This systematic review and meta-analysis were prospectively registered with PROSPERO and adhered to both PRISMA (preferred reporting for systematic reviews and meta-analyses) and MOOSE (meta-analysis of observational studies in epidemiology) recommendations [34, 39, 56].

### Systematic search strategy

Systematic searches of the peer-reviewed literature was conducted following PRESS guidelines [47] in consultation with a medical librarian in four electronic databases (i.e., MEDLINE, PsycINFO, Embase, and CINAHL) from inception to October 13th, 2021. The key words included two concepts: (1) anorexia nervosa (AN) and (2) substance Use or substance use disorder terms. Database searches and an exhaustive list of key terms are provided in the Additional file 1: search material. Two blinded reviewers performed title/abstract screening (A.A. and J.F.) and full-text article screening (A.A. and A.S.) in duplicate. In addition, reference lists of included articles were hand-searched for other relevant studies.

### Study selection criteria

Two reviewers selected peer-reviewed articles (A.A. and A.S.) for inclusion in this systematic review based on the following criteria: (1) research including participants with anorexia nervosa (AN), restrictive AN (AN-R), and AN of the binge-eating/purging type (AN-BP); and (2) reported on the prevalence of either substance use (e.g., alcohol use, tobacco use, cannabis use), substance use disorders, or drug abuse/misuse/dependence disorders. In addition, this review excluded studies that: (1) looked at the relationship between AN and other behavioral addictions (e.g., gambling disorder) or impulse control disorders, (2) study designs that were case reports, review articles, opinion pieces, and editorials, (3) studies that included use/abuse of prescribed medications and (4) did not report sufficient information to calculate a prevalence rate. Disagreements were first discussed in a consensus meeting, and D.D. decided on inclusion or exclusion.

### Data extraction

Data extraction for Table 1 was completed in duplication (A.A. and G.P.), including the following study and participant characteristics: author, year of publication, country, study type, types of substance use/abuse/dependence, AN types, age (mean ± SD), percent female (number of females), and outcomes reported. For the meta-analysis, the following data were extracted in duplicate (A.A. and D.D.): (1) author, (2) year of publication, (3) types of substance use, substance abuse/dependence disorders, and drug abuse/dependence disorders, (4) AN type, (5) numerator representing substance use/abuse/dependence disorders, and drug abuse/dependence disorders, (6) denominator representing AN sample, and (7) lifetime prevalence or period prevalence.

**Table 1:** Details of Included Studies (n = 52)

Study	Year	Country	Type(s) of SUDs or substance use	Type(s) of AN	AN patients		
					N	Age (Mean ± SD)	% Female
Anzengruber et al.	2006	USA	Smoking (nicotine)	AN-R, AN-P, AN-B, ANBN	AN-R = 306, AN-P = 186, AN-B = 107, ANBN = 180	Initial sample (n = 897): 26.3 ± 8.3	100%
Blinder et al.	2006	USA	Alcohol, cannabis, polysubstance, other substance, amphetamine, sedative/hypnotic/anxiolytic, cocaine, hallucinogen, opioid, inhalant abuse/ dependencies	AN-R, AN-B	AN-R = 520, AN-B = 436	AN-R = 20.9 ± 9.1 AN-B = 23.8 ± 8.5	100%
Bodell et al.	2013	USA	SUD	AN	30	Total sample: 35 $\pm$ 9	100%
Braun et al.	1994	USA	Alcohol/substance dependence	AN-R	34	24.8	100%
Bulik et al.	1992	USA	Licit and illicit substance use	AN	27	20.3 $\pm$ 10.5	100%
Bulik et al	2008	USA	Psychoactive substance abuse and dependence	AN-R, AN-P, AN-B, ANBN	432	30.4 $\pm$ 11.3	95% (n = 410)
Burgalassi et al.	2009	Italy	Caffeine	AN-R, AN-BP	15	26 ± 5	100%
Carlat et al.	1997	USA	Substance abuse	AN	30	At onset: 19.0 $\pm 5.6$ At first treatment: 20.3 $\pm 6.0$	0%
Casper and Jabine	1996	USA	Alcohol abuse/dependence, drug abuse/dependence, tobacco dependence	AN-R, AN-P	75	Early Adolescent Onset: 16.2 $\pm 3.3$ Late Adolescent Onset: 19.7 $\pm 3.3$ Adult Onset: 25.2 $\pm 3.7$	100%
Corbridge and Bell	1996	UK	Alcohol and drug misuse	AN	25	Age of onset: 23.2	Total sample: 97% (n = 125)
Corcos et al.	2001	France, Switzerland, Belgium	Alcohol, drug, and psychotropic consumption	AN-R, AN-P	AN-R = 111, AN-P = 55	AN-R = 19.3, AN-P = 20.6	100%
Deter and Herzog	1994	Germany	Substance abuse	AN-R, AN-P	AN-R = 29, AN-P = 55	Presentation: 20.7 $\pm 6.0$ Follow-up: 32.5 $\pm 6.1$	100%
Eddy et al.	2002	USA	Drug and alcohol abuse	AN-R, AN-BP	AN-R = 51, AN-BP = 85	AN-R (no B/P history) = 20.8, AN-R (history of B/P) = 23.8, AN-BP = 22.7	100%
Fairburn et al.	1999	UK	Drug and alcohol abuse	AN	67	22.4 $\pm$ 4.8	100%
Fichter and Quadlieg	1999	Germany	Substance abuse (including alcohol and tranquilizers)	AN-R, AN-BP	AN-R = 30, AN-BP = 73	Upon admission: 24.9 $\pm 6.7$	100%
Fichter et al.	2006	Germany	Substance abuse (including drug dependence)	AN-R, AN-BP	AN-R = 30, AN-BP = 73	Upon admission: 24.9 $\pm 6.7$	100%
Fioravanti et al.	2014	Italy	Cocaine and amphetamine abuse	AN-R, AN-BP	AN-R = 28, AN-BP = 35	AN-R: 25.93 $\pm 8.94$ AN-B/P: 25.77 $\pm 8.80$	100%
Franko et al.	2005	USA	Alcohol Use Disorder (AUD)	AN-R, AN-BP	AN-R = 51, AN-BP = 85	Not reported (Inclusion: > 12 years)	100%
Franko et al.	2008	USA	Drug Use Disorder (DUD)	AN-R, AN-BP	AN-R = 51, AN-BP = 85	Entire sample at entry: 24.68 $\pm 6.7$	100%
George and Waller	2005	UK	Smoking/nicotine	AN	25	31.2 ± 2.16	100%
Hall et al.	1984	New Zealand	Alcohol Abuse Disorder	AN	50	Onset: 16.2 $\pm 2.7$ Presentation: 20.1 $\pm 5.6$	100%
Haug et al.	2001	USA	Tobacco, caffeine, alcohol, marijuana, other drug use (cocaine, opiates, hallucinogens)	AN-R, AN-P	AN-R = 34, AN-P = 31	Total sample: 26.2 ± 11.5	100%
Henzel	1984	USA	Alcoholism	AN	15	24 (median)	80% (n = 12)
Herzog et al.	1992	USA	SUD	AN, ANBN	AN = 41, ANBN = 90	AN = 19.1 ± 6.4, ANBN = 17.5 ± 4.6	100%
Herzog et al.	1999	USA	SUD	AN-R, AN-BP	AN-R = 51, AN-BP = 85	AN-R = 23.9 ± 8.5, AN-BP = 24.5 ± 5.9	100%
Herzog et al.	2006	USA	DUD; narcotics, amphetamines, cocaine, sedatives, marijuana, LSD, solvents, polydrug use	AN-R, AN-BP	AN-R = 51, AN-BP = 85	Not reported (Inclusion: > 12 years)	100%
Hudson et al.	1983	USA	Alcohol abuse/dependence, Amphetamine abuse or dependence, Other substance use	AN ANBN	AN = 16 ANBN = 25	AN = 25.0 ± 7.0 (15 Females only, one male aged 24) ANBN = 25.8 ± 7.3 (24 females only, one man aged 28)	95.1% (n = 39)
Iwasaki et al.	2000	Japan	Alcohol abuse, Sedative abuse, Inhalant abuse	AN-R AN-BP	AN-R = 62 AN-BP = 36	AN-R = 21.3 ± 5.5 AN-BP = 24.2 ± 4.7	100%
Jordan et al.	2003	New Zealand	Alcohol abuse/dependence, cannabis abuse/dependence, psychoactive SUD	AN-R, AN-BP	AN-R = 24, AN-BP = 16	23.15 ± 6.69	100%
Jordan et al.	2008	New Zealand	Alcohol abuse/dependence, cannabis abuse/dependence, any psychoactive SUD	AN-R, AN-BP	AN-R = 31, AN-BP = 25	20.5	100%
Kask et al.	2016	Sweden	Alcohol use disorder, other SUD	AN	8069	19.5 ± 6.2	100%
Kask et al	2017	Sweden	Alcohol use disorder, other substance use disorder	AN	609	Mean age of first hospitalization: 18.2 ± 6.9	0%
Kirkpatrick et al.	2019	Canada	Substance Use	AN-R, AN-BP, atypical AN	AN-R = 40, AN-BP = 19, Atypical AN = 14	SUG (all ED): 16.3 $\pm 1.0$ NSUG (all ED): 14.3 $\pm 2.9$	SUG (all ED) 97.6% NSUG (all ED) 81.7%
Krahn et al.	1991	USA	Caffeine consumption	AN, ANBN	AN = 14, ANBN = 11	Total sample: 24.7	N/A
Krug et al.	2008	Spain, UK, Italy, Austria, Slovenia	Tobacco, alcohol, and drug use	AN-R, AN-BP	AN-R = 172, AN-BP = 156	All EDs: 27.2 $\pm$ 8.9	All EDs: 96.6% (n = 849)
Laessle et al.	1989	Germany	SUD	AN-R, AN-BP	AN-R = 21, AN-BP = 20	AN-R = 20.9 ± 4.1, AN-BP = 22.1 ± 4.2	100%
Machado et al.	2004	Portugal	Alcohol and drug abuse	AN-R, AN-BP	AN-R = 42, AN-BP = 23	16.8 $\pm 13.4$	100%
Mann et al.	2014	USA	Substance use (alcohol, cannabis, tobacco, and any other substance)	AN	118	Total sample: 15.77 ± 1.84	Total sample: 90.7%
Milos et al.	2003	Switzerland	Substance related disorders (use and dependency)	AN	77	24.7 ± 5.6	100%
Nagata et al.	2000	Japan	Alcohol and Illicit drug (heroin, amphetamines, other psychoactive substances) use	AN-R, AN-BP	AN-R = 60, AN-BP = 62	AN-R = 22.3 ± 4.0, AN-BP = 25.0 ± 5.1	100%
Nagata et al.	2002	Japan	DUD	AN-R, AN-BP	AN-R = 62, AN-BP = 48	ED and DUD: 25.1 ± 5.7 ED without DUD: 24.2 ± 5.5	100%
Nagata et al.	2003	Japan	Drugs other than alcohol	AN-BP, AN-R	AN-BP = 8, AN-R = 2	Total ED + DUD group: 24.8 ± 5.4	100%
Nozoe et al.	1995	Japan	Stimulant (alcohol, coffee, cigarettes) abuse	AN	55	Total sample at admission: 20.0	90.9% (n = 50)
Selby et al.	1995	USA	Substance misuse problems	AN	25	Entire ED sample: 30.07 ± 13.78	100%
Strober et al.	1995	USA	Alcohol, cocaine, amphetamines, cannabis, polysubstance use/abuse	AN-R, AN-B	AN-R = 77, AN-B = 18	15.1 (SD not reported)	94% (n = 89)
Sullivan et al.	1998	New Zealand	Alcohol, cannabis, other drug, and any drug dependence	AN	70	At admission: 20.9 $\pm 8.0$ Interview: 32.4 $\pm 7.8$	100%
Tanaka et al.	2001	Japan	Alcohol abuse	AN-R, AN-BP	AN-R = 27, AN-BP = 34	At first referral: 22.7 ± 6.0 years	100%
Toner et al.	1986	Canada	SUD; alcohol, drug, and tobacco abuse	AN-R, AN-B	AN-R = 30, AN-B = 25	AN-R = 28.0 $\pm$ 5.3 AN-B = 28.2 $\pm 4.0$	100%
Ulfvebrand et al.	2015	Sweden	Alcohol dependence, Alcohol abuse, Substance dependence, Substance abuse, Substance use disorders	AN-R AN-BP	AN-R = 926 AN-BP = 466	AN-R (female) = 24 AN-R (male) = 23 AN-BP (female) = 24 AN-BP (male) = 21 (SD not reported)	96.7% (n = 6921)
Wiederman and Pryor	1996A	USA	Alcohol, amphetamines, barbiturates, hallucinogens, marijuana, tranquilizers, cocaine, cigarettes	AN	134	Total sample: 24.15 ± 7.69	100%
Wiederman and Pryor	1996B	USA	Alcohol, amphetamines, barbiturates, hallucinogens, marijuana, tranquilizers, cocaine, cigarettes	AN-R, AN-BP	AN-R = 46, AN-BP = 13	Total sample: 15.44 ± 1.35	100%
Wiseman et al.	1998	USA	Cigarettes (nicotine)	AN-R, AN-BP	AN-R = 56, AN-BP = 12 (included with BN group)	Total ED group: 15.35 ± 1.4	100%

*AN-R* Anorexia nervosa-restricting type; *AN-P* anorexia nervosa-purging type; *AN-B* anorexia nervosa-binging type; *ANBN* anorexia nervosa and bulimia nervosa; *ED* eating disorder; *AN-BP* anorexia nervosa, binge-eating/purging type; *BN* bulimia nervosa; *MDD* major depressive disorder; *AUD* alcohol use disorder; *DUD* drug use disorder; *SUG* substance use group; *NSUG* no substance use group

### Risk-of-bias assessment

Studies included in this systematic review and meta-analysis were assessed for quality using a modified Downs and Black instrument [19] which contains 14-items for cross-sectional studies, providing a total score of 15 for each study indicating greater quality. Scores of ≥ 11.5 (> 75%), 9–11 (60–74%), and < 9 (< 60%) were taken to indicate high, moderate, and low quality, respectively.

### Data synthesis and analysis

Due to potential heterogeneity between studies, successions of DerSimonian and Laird [18] random-effects meta-analyses were performed on eligible studies to estimate the pooled prevalence and 95% CIs for substance use disorders, drug abuse/misuse/dependence disorders, and substance use. The primary outcome measure was total substance use disorders and the summary statistic used in the meta-analysis was the pooled prevalence. Many studies distinguish total substance use disorders from total drug abuse/dependence disorders by not including alcohol in the drug abuse/dependence disorder count. Thus, these two concepts were kept separate in the meta-analysis. In addition, differences in the pooled prevalence between AN-R and AN-BP were examined. All meta-analyses in this paper employed Freeman-Tukey double arcsine transformations, with the exact confidence interval method, by computing the weighted pooled estimate and then performing a back-transformation on the pooled estimate. This approach is favorable where there is zero count prevalence as it prevents these studies from being dropped from the meta-analysis, which would create a bias in prevalence estimates. Lifetime prevalence and period prevalence were first examined separately, but there was minimal variation between the two prevalence types. Thus, we combined the two (e.g., some studies reported lifetime and other studies reported period prevalence, both types were included in the same meta-analysis) to provide an overall prevalence for each outcome. Two studies was the minimum amount of studies included in each pooled meta-analysis. However, prevalence was also presented when reported by just one study, however this is not an estimate derived from a meta-analysis. Statistical heterogeneity was examined using the I^2^ statistics, an I^2^ value is only produced for a meta-analysis with four or more studies in the meta-analysis. We performed all analyses in STATA v.17 [54] and produced forest plots showing the prevalence of those with either substance use disorders or drug abuse/dependence disorders in those with AN.

## Results

### Search yield

**Fig. 1:**
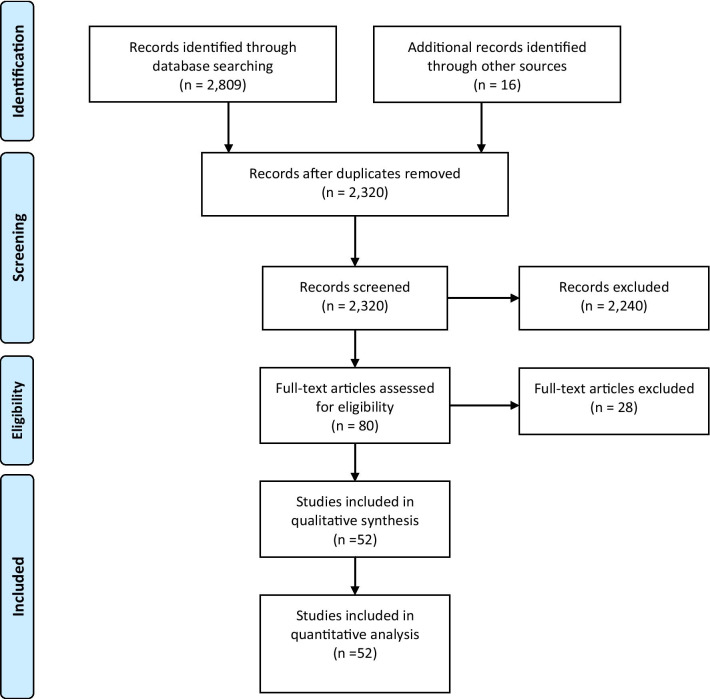
PRISMA flow diagram

Database searches returned 2809 abstracts and titles. After duplicate references were removed, 2320 abstracts and titles were screened. The level of agreement between two blinded reviewers for screening was moderate (*κ* = 0.65). After resolution of discrepancies, eighty full-text studies were retrieved and reviewed independently, of which a total of 52 studies met the inclusion criteria for this review (see Fig. 1). In total, 35 studies measured SUDs, 17 measured substance use, with six of these studies measuring both SUDs and substance use.

### Participant characteristics and study characteristics

There was a total 14,695 individuals identified as having AN included in this review, ranging from sample sizes of 15 to 8069 for individuals with AN in separate studies, Table 1. The mean age of individuals with AN was 22.82 years (Range 14.3–35.0), and the percentage of females was 95.6%.

Studies were published between 1983 and 2019. Most studies were conducted in North America (n = 26), followed by Europe (n = 16), Asia (n = 6), and New Zealand (n = 4). Twenty-two studies included a comparison or control group in their respective study. Forty-two studies identified individuals with AN from a hospital setting (9 = outpatient, 7 = inpatient), ED programs, or specialized clinic, with six studies identifying AN individuals from research studies. Additional file 1: Table S2 describes in greater detail outcomes reported in each study, including subgroup analyses, comparison groups, sex differences, and other relevant information related to AN and SUDs or substance use outcomes.

### Quality assessment of included studies

All studies included in this systematic review were evaluated with the modified Downs and Black instrument (Additional file 1: Table S1). The average Downs and Black score was 10.9/15, demonstrating mostly moderate quality across studies. The majority of studies clearly described their main aims, measures, and findings. However, most studies included in this review failed to account for the effects of significant covariates.

### Prevalence of substance use disorders and drug abuse/dependence

**Fig. 2:**
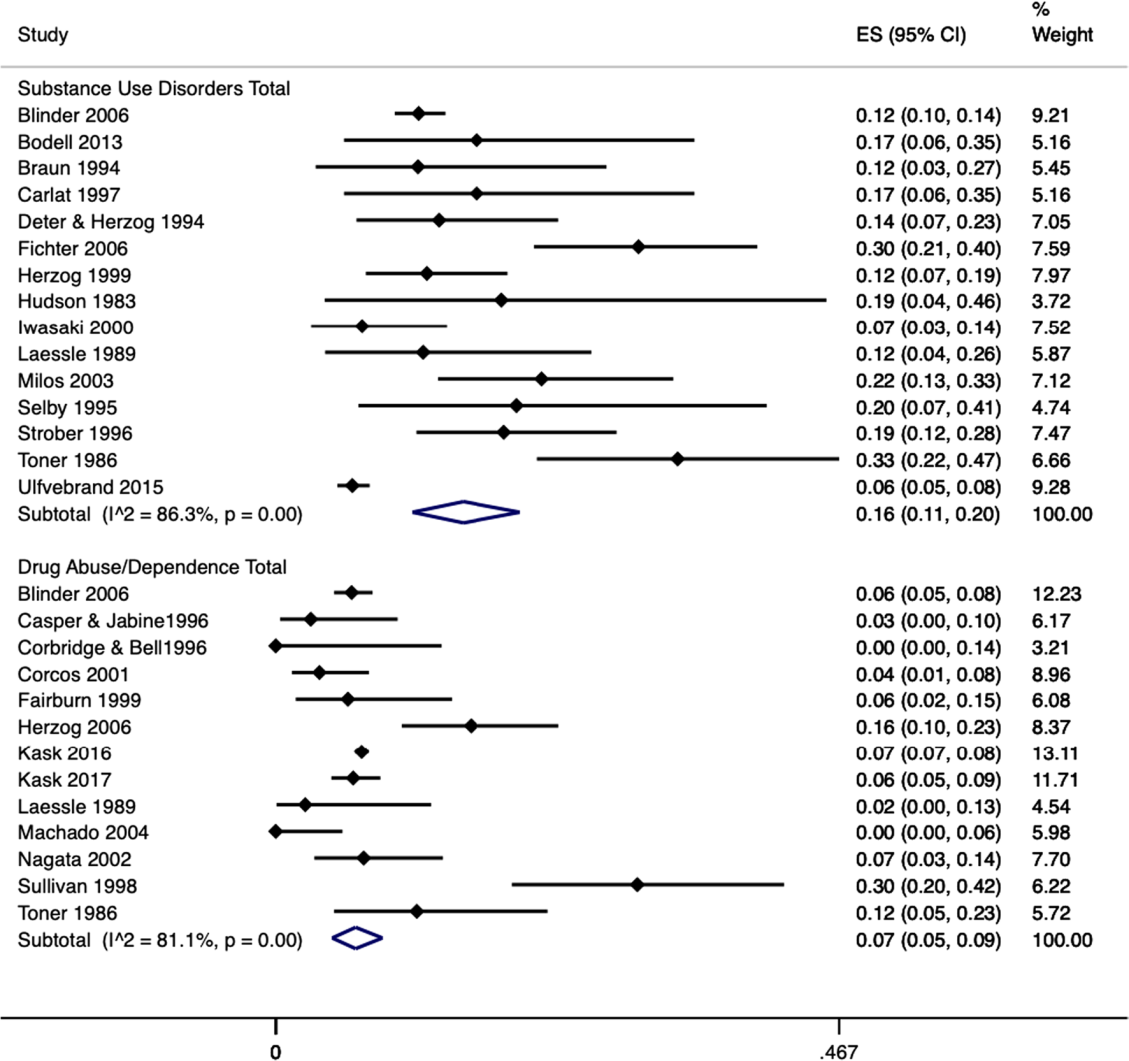
Forest plot of the prevalence of substance use disorders and drug abuse/dependence disorders in AN. Notes: Blue diamond represents the overall pooled effect per outcome, ES = effect size

In studies that looked at total substance use disorders, random pooled estimates demonstrated that substance use disorders had a 16% prevalence in those with AN (95% CI = 0.11–0.20; I^2^ = 86.3%; 15 studies, N = 3118), see Fig. 2. Individuals with AN-BP had a higher prevalence of substance use disorders at 18% (95% CI = 0.12–0.26; I^2^ = 82.1%; 6 studies, N = 1058) compared to 7% in those with AN-R (95% CI = 0.04–0.10; I^2^ = 69.3%; 8 studies, N = 1635), see Additional file 1: Figure S1.

Drug abuse/dependence disorders had a prevalence of 7% in AN (95% CI = 0.05–0.09; I^2^ = 81.1%; 13 studies, N = 10,443), with individuals with AN-BP having a higher prevalence of drug abuse/dependence at 9% (95% CI = 0.03–0.17; I^2^ = 65.8%; 5 studies, N = 235) compared to 5% in those with AN-R (95% CI = 0.02–0.09;; I^2^ = 35.8%; 5 studies, N = 278), see Additional file 1: figure S2.

### Prevalence of specific substance use disorders

In studies that looked at specific substance use disorders, random pooled estimates demonstrated that there was a 10% prevalence of alcohol abuse/dependence in AN (15% AN-BP vs. 3% AN-R), 6% prevalence of cannabis abuse/dependence in AN (4% AN-BP vs. 0% AN-R), and a 5% prevalence of amphetamine abuse/dependence in AN. However, the majority of specific substance use disorders identified in this review remained low comparatively. Prevalence estimates for other specific forms of substance abuse/dependence are provided in Table 2.

**Table 2:** Prevalence of substance use disorders in AN, AN-R, and AN-BP

Substance use disorder (abuse/dependence)	*k*	*n*	*I*^2**^	95% CIs	Prevalence in AN	n for AN-R	Prevalence in AN-R	n for AN-BP	Prevalence in AN-BP
Alcohol	25	12,655	84.08%	0.08,0.13	10%	1752	3%	1138	15%
Cannabis	6	1379	88.65%	0.02,0.12	6%	551	0%	461	4%
Polysubstance	3	1187		0.01,0.04	3%	520	2%	436	4%
Amphetamine	4	1203	92.79%	0.00,0.15	5%	520	0%	436	0%
Sedative/hypnotic	3	1190		0.00,0.03	1%	582	0%	472	1%
Cocaine	5	1280	83.02%	0.00,0.08	3%	548	0%	471	1%
Hallucinogen	2	1092		0.00,0.01	0%	520	0%	436	0%
Opioid	1*	956		0.00,0.01	0%	520	0%	436	0%
Inhalant	2	1054		0.00,0.00	0%	582	0%	472	0%
Narcotic	1*	136		0.00,0.05	1%	N/A	N/A	N/A	N/A
Other substances	4	1098	88.92%	0.00,0.14	4%	551	0%	461	0%
Substance Use Disorder Total	15	3118	86.3%	0.11,0.20	16%	1635	7%	1058	18%
Drug Abuse/ Dependence Total	13	10,443	81.1%	0.05,0.09	7%	278	5%	235	9%

^*^not derived from mete-analysis data, ** ***I***^**2**^ only derived when pooled meta-analysis included 4 or more studies, *I*^2^ for AN prevalence

*AN* anorexia nervosa; *AN-BP* anorexia nervosa binge-eating/purge type; *AN-R* anorexia nervosa restrictive type; *CIs* confidence intervals; *k* amount of studies

### Prevalence of substance use

There was a 20% prevalence for substance use (95% CI = 0.08–0.34; I^2^ = 94.27; 4 studies, N = 769) in studies that looked at substance use total in individuals with AN. In studies that looked at types of substance use in individuals with AN, there was a 37% prevalence for caffeine (95% CI = 0.08–0.73; 3 studies, N = 107), followed by a 29% prevalence for alcohol (95% CI = 0.22–0.36; I^2^ = 70.56; 5 studies, N = 677), 25% for tobacco (95% CI = 0.16–0.34; I^2^ = 90.65; 9 studies, N = 1352), 14% for cannabis (95% CI = 0.03–0.28; I^2^ = 95.18; 5 studies, N = 720), and 14% stimulants use (95% CI = 0.11–0.18; 2 studies, N = 383). However, the majority of specific substance use identified in this review remained low comparatively. Prevalence estimates for other forms of substance use are provided in Table 3.

**Table 3:** Prevalence of Substance Use in AN, AN-R, and AN-BP

Prevalence									
Substance Use	*k*	*n*	*I*^2 **^	95% CIs	Prevalence in AN	n for AN-R	Prevalence in AN-R	n for AN-BP	Prevalence in AN-BP
Alcohol	5	677	70.56%	0.22,0.36	29%	172	31%	156	43%
Amphetamines	3	326		0.01, 0.06	3%	N/A	N/A	N/A	N/A
Barbiturates	2	160		0.00, 0.05	2%	N/A	N/A	N/A	N/A
Caffeine	3	107		0.08, 0.73	37%	N/A	N/A	N/A	N/A
Cannabis	5	720	95.18%	0.03, 0.28	14%	283	16%	211	33%
Cocaine	3	326		0.00, 0.04	1%	111	0%	55	0%
Diazepam	1*	26		0.00, 0.20	4%	N/A	N/A	N/A	N/A
Hallucinogens	2	161		0.00, 0.02	0%	N/A	N/A	N/A	N/A
Heroin	1*	166		0.00, 0.02	0%	N/A	N/A	N/A	N/A
Inhalants	2	148		0.00, 0.04	1%	N/A	N/A	N/A	N/A
Opiates	3	521		0.00, 0.24	5%	111	1%	55	2%
Phencyclidine	1*	26		0.00, 0.13	0%	N/A	N/A	N/A	N/A
Quaaludes	1*	26		0.00, 0.20	4%	N/A	N/A	N/A	N/A
Stimulants	2	383		0.11, 0.18	14%	N/A	N/A	N/A	N/A
Tobacco	9	1352	90.64%	0.16, 0.34	25%	255	26%	788	32%
Tranquilizers	1*	134		0.00, 0.06	2%	N/A	N/A	N/A	N/A
Other Substance Use	1*	328		0.10, 0.18	14%	N/A	N/A	N/A	N/A
Substance Use Total	4	769	94.27%	0.08, 0.34	20%	111	5%	55	18%

^*^not derived from meta-analysis data, ** ***I***^**2**^ only derived when pooled meta-analysis included 4 or more studies

*AN* anorexia nervosa; *AN-BP* anorexia nervosa binge-eating/purge type; *AN-R* anorexia nervosa restrictive type; *CIs* = confidence intervals; *k* amount of studies

## Discussion

### Summary of findings

The present meta-analysis evaluated the prevalence of co-occurring substance use and substance use disorders (SUD) among persons with anorexia nervosa (AN). In total, 52 studies met review eligibility criteria. The overall prevalence of substance use of any kind was 20%, including caffeine (37%), alcohol (29%), tobacco (25%), cannabis (14%), and stimulants (14%). The overall prevalence of any SUD was 16%, including drug abuse/dependence (7%), alcohol (10%), cannabis (6%), and amphetamines (5%). Globally, the prevalence of drug use disorders among the population aged 15–64 is estimated to be 0.71% (Drugs & Crime, 2019), thus the prevalence in AN appears to be high comparatively. The sample included in this meta-analysis was predominantly female, however, when looking at studies that included only males in the current review, males had an estimated prevalence of 17% for SUD total, 6% for drug abuse/dependence, and between a 8–13% prevalence for alcohol abuse/dependence, which appears to be similar to females but based on very few studies. The overall sample was also younger in this meta-analysis with a mean age of 23, and potentially overtime the prevalence of these disorders may increase. Substance use and SUD prevalence were higher among people with the binge-eating/purge (AN-BP) type than those with the restrictive (AN-R) type throughout. However, the majority of SUDs and substance use appear to be very low or not present in AN patients including sedative use, hallucinogen use, opioids, and inhalants.

### Treatment implications

The high prevalence of comorbid substance use and SUD in persons with AN has important treatment implications. SUD management in the absence of ED comorbidity begins with a thorough psychiatric interview for diagnostic evaluation and treatment planning [35]. Stress and trauma are etiological factors in both SUD and ED, and diagnostic interviews must consider these aspects [12]. Treatments for ED and SUD are individualized, substance- or ED-specific, and mindful of an individual’s intrinsic motivation and readiness for change [62]. For SUD, individuals identify abstinence or harm reduction goals; the latter refers to continued substance use instead of incrementally using less to mitigate risk [31]. With these general frameworks in mind, the next component of treatment is usually tailored towards the specific ED-SUD combination. A variety of psychotherapeutic, psychosocial, and even pharmacological therapies are available for ED and SUD, but few studies have explored treatments for co-occurring disorders [6]. At present, clinicians generally pick treatments that could work synergistically, as some therapies have indications for both diseases. For alcohol use disorder (AUD), the three first-line medications are naltrexone (an opioid receptor antagonist blocking the endogenous reward associated with alcohol consumption), disulfiram (an inhibitor of acetaldehyde dehydrogenase, causing a toxic reaction if alcohol is consumed due to accumulation of alcohol metabolites), and acamprosate (an NMDA receptor antagonist that reduces cravings for alcohol). For opioid use disorder (OUD), first-line pharmacotherapies include methadone and buprenorphine, synthetic opioids that suppress opioid withdrawal and cravings [4, 32]. For tobacco use disorder (TUD), nicotine replacement therapy, varenicline (a partial agonist of the acetylcholinergic receptor), and bupropion (a noradrenergic-dopaminergic antidepressant) are evidence-based treatments that can improve quit rates and sustained abstinence from tobacco [37]. While the DSM-5 does not currently recognize caffeine use disorder, caffeine withdrawal and intoxication are formal diagnoses [1]. Generally, it is possible that treatments for co-occurring SUD in people with AN may not interfere with AN treatment.

Given the high degree of comorbidity between SUDs and AN, it is essential to develop treatment strategies that are effective for both conditions. Pharmacologically, most medications used to treat AN or to treat SUD are compatible with one another. For example, the use of opioid agonist therapies for opioid use disorder and naltrexone or acamprosate for alcohol use disorder could complement the pharmacological treatment. However, as some of these medications for SUDs can prolong the QTc interval, they raise the risk of new-onset cardiac arrhythmias, which may be more likely in persons with AN who are very underweight and if they have electrolyte abnormalities. There are currently no approved pharmacological interventions for stimulant, cannabis [5, 7], or hallucinogen use disorders. For cannabis use disorder (CUD), the occurrence of cannabis withdrawal and intoxication can also induce anorexia symptoms, nausea, vomiting, and weight loss and can occur in nearly half of persons with CUD [8]. For CUD, there are no approved pharmacotherapies, and the only treatment with current efficacy for withdrawal symptoms is sustained abstinence.

Nonetheless, several psychosocial interventions have evidence for both ED and co-occurring SUD, such as self-help approaches [59], mindfulness-based cognitive behavioral therapy [15], dialectical behavioral therapy [14], family and couples therapy [42], and contingency management [20]. While there are several reasons why some psychosocial interventions may demonstrate efficacy for co-occurring AN and SUD, one reason might be transdiagnostic psychopathology that responds to the same types of treatments [36]. For example, Claudat et al. made a case for the effectiveness of DBT for persons with co-occurring EDs and SUD as such persons share difficulties with emotion regulation, goal-directed activity, and impulsivity [14].

New and innovative interventions such as real-time fMRI-based neurofeedback, transcranial magnetic stimulation, transcranial direct current stimulation, and deep brain stimulation aim to influence brain regions' activity to regulate food and addictive substance intake [17]. These interventions follow the assumption that there are two circuits in the brain controlling both food intake in EDs and substance use in SUDs; the first circuit responds to salient/rewarding stimuli and consists of structures like ventral striatum, amygdala, anterior insula, ventromedial prefrontal cortex/orbitofrontal cortex. The second circuit regulates the degree of cognitive control over food or addictive substance intake and includes brain structures such as the anterior cingulum and dorsolateral prefrontal cortex [58]. Thus, future research in the form of randomized control trials are needed to test the variety of treatment strategies mentioned above in an effort to reduce symptoms in individuals suffering from comorbid SUDs and AN.

### Overlapping features between SUDs and AN

Although SUD and AN potentially appear to respond to some of the same treatments, these disorders are distinct. However, both conditions are behaviourally defined psychiatric disorders [46] and may also have some genetic overlap [41]. For SUD, aberrations in the endogenous reward system drive pathological craving and drug-seeking behaviors, leading to a continued cycle of intoxication and withdrawal [2]. For AN, a negative view of one’s body image drives caloric restriction, low BMI, and for some, repeated compensatory behaviors to maintain low body weight, such as purging, extreme exercise, laxative use, and fasting, often associated with subjective and objective binge episodes [1]. Several working hypotheses have been put forward as potential explanations for their overlap as these two disorders commonly co-occur [25]. The first hypothesis involves self-regulation through self-medication, which means that substance use and AN behavior (e.g., restriction, binge behaviors, purging) are used to “treat” an underlying pathology [30, 52]. Frequently, persons with SUD and AN both describe a chaotic inner milieu that temporarily abates from the effects of substances or caloric restriction [9]. The second hypothesis assumes shared risk factors or underlying causes. For example, a drive towards perfectionism, impulsivity, novelty-seeking, and rigidity/obsessions appear to raise the risk for the development of both ED and SUD [38, 53]. One of our review’s findings -that the prevalence of substance use and SUD was higher in AN-BP compared to AN-R- appears to support this second hypothesis, as AN-BP has more features consistent with the impulsive, novelty-seeking phenotype seen in persons with SUD [11, 43, 50]. Finally, specific substances may appear to serve a functional purpose for forms of ED. In the setting of ED, appetite-suppressing substances, such as tobacco, may help maintain a low appetite [3, 40]. In addition, caffeine and stimulants may also suppress appetite, maintain caloric restriction, fuel intense exercise, and address fatigue stemming from diminished BMI [10, 55]. Alcohol may lessen the severity of AN-induced anxiety and affective symptoms [13] and may increase appetite [51].

Recent neurobiological findings support the notion that mechanisms of addiction may also be involved in the development and maintenance of AN [44, 61]. On the one hand, high-calorie food cues, which may be considered “incompatible” with AN, are processed with anxiety and associated with increased activation of brain regions responsible for inhibitory control [61], which is per avoidance bias regarding food found in individuals with AN [45]. On the other hand, disorder-compatible stimuli (e.g., images of underweight women's bodies, physical activity cues) are appetitively processed; such sensitization processes of the reward system may lead to maintaining the problematic behavior patterns seen in AN. For example, females with AN instructed to imagine that their own body would correspond to specific normal-weight or underweight body cues showed more robust activation in structures of the reward system, particularly in the ventral striatum, during the self-referential processing of images of underweight bodies compared with normal-weight bodies; the opposite pattern was found for healthy female subjects [23]. Similar results were shown using other techniques as well: both EEG and eye-tracking studies, as well as studies in which the blink reflex was recorded as a measure of appetitive valence, have revealed an attentional bias/positive processing for images of underweight female bodies and images of physical activity [27], comparable to the processing of alcohol-associated stimuli in alcohol-dependent patients. Accordingly, O'Hara and colleagues [44] postulated a "reward-centered" model of AN, which assumes that food-associated stimuli are experienced as aversive.

In contrast, disorder-compatible stimuli (such as underweight body images and physical activity) are processed positively and activate the mesolimbic reward system. Also, individuals with AN exhibit greater activation in prefrontal brain areas, somatosensory cortex, and cerebellum when responses to physical activity stimuli are to be inhibited in a go-no-go task [33]. This also corresponds to findings regarding the neuronal activation patterns of alcohol-dependent patients during response inhibition towards alcohol-associated stimuli. Such findings suggest an inhibition deficit for disorder-compatible rewarding behaviors.

### Strengths and limitations

The present meta-analysis has several strengths. The robust methods and adherence to PRESS, PRISMA, and MOOSE guidelines are one, while the large yield of studies (n = 52) and participants (n = 14,695 individuals identified as having AN) were others. In addition, the studies included in this meta-analysis were of fair to moderate quality. The review also advances the field by focusing on the prevalence of both substance use and SUD in persons with AN. While a previous review by Bahji et al. found similar SUD prevalence estimates in EDs [6], the present study identified more studies. While having SUD estimates provides a meaningful assessment of clinically significant impairment, the additional information provided by substance use helps contextualize the specific patterns of substances that are more likely to lead to functional consequences in persons with AN.

However, there are a few limitations. First, as a meta-analysis of prevalence, we encountered high heterogeneity when pooling estimates across studies. Some of this heterogeneity occurred from combining the different types of AN, and stratification into AN-BP and AN-R-specific estimates helped reduce some heterogeneity. However, there are other potential sources of heterogeneity that we did not explore analytically due to the limited number of studies per subgroup analysis. For example, variations in substance use and SUD measurements across studies likely increased heterogeneity due to different ascertainment methods (e.g., self-report, informant-report, structured interviews, urine drug screens) and alternative diagnostic criteria (e.g., DSM-III, DSM-IV, and DSM-5). In addition, many specific substance use disorders and different types of substance use had a very low prevalence in those with AN, however this may be due to the limited amount of studies available for these outcomes. Specifically, many of the substance use prevalence rates were much lower than the general populations, such as alcohol use and caffeine use. The generalizability of the results is another limitation, as most participants were young women; consequently, our review’s findings are less applicable to males in general and older populations. Finally, we cannot determine causal relationships between substance use, SUD, and AN as an observational review.

### Conclusions

This is the most comprehensive meta-analysis on the comorbid prevalence of SUDs and substance use in persons with AN, with an overall pooled prevalence of 16%. Comorbid SUDs were much more common in AN-BP compared to AN-R. Clinicians should be aware of the high prevalence of specific SUD comorbidity and substance use in individuals with AN. Finally, clinicians should consider screening for SUDs and integrating treatments that target SUDs in individuals with AN.

## Supplementary Information

**Additional file 1**. Supplementary Review Material.

## Data Availability

Not applicable.

## Abbreviations

AN-R: Anorexia nervosa-restricting type
AN-P: Anorexia nervosa-purging type
AN-B: Anorexia nervosa-binging type
ANBN: Anorexia nervosa and bulimia nervosa
ED: Eating disorder
AN-BP: Anorexia nervosa, binge-eating/purging type
BN: Bulimia nervosa
MDD: Major depressive disorder
AUD: Alcohol use disorder
DUD: Drug use disorder
SUG: Substance use group
NSUG: No substance use group
