# Supplementary material for: The prevalence of substance use disorders and substance use in anorexia nervosa: a systematic review and meta-analysis
Source: J Eat Disord. 2021 Dec 11;9:161. doi: 10.1186/s40337-021-00516-3 (PMC8666057; doi:10.1186/s40337-021-00516-3)
Supplement: Supplementary file 1 — Additional file 1. Supplementary Review Material. [file 40337_2021_516_MOESM1_ESM.docx]

**Supplementary Material:** Searches

** searches first conducted on January 28, 2021 and then updated on October 13^th^, 2021.

Ovid MEDLINE(R) and Epub Ahead of Print, In-Process, In-Data-Review & Other Non-Indexed Citations, Daily and Versions(R)

| **#** | **String** | **Yield** |
| --- | --- | --- |
| 1 | exp "Feeding and Eating Disorders"/ | 31354 |
| 2 | (anorex* nervosa or bulim* nervosa or binge* eat* or purg*).mp. | 31401 |
| 3 | (eating adj4 disorder*).mp. | 29930 |
| 4 | (feeding adj4 disorder*).mp. | 16956 |
| 5 | (appetite adj4 disorder*).mp. | 751 |
| 6 | OSFED.mp. | 48 |
| 7 | ARFID.mp. | 154 |
| 8 | 1 or 2 or 3 or 4 or 5 or 6 or 7 | 51147 |
| 9 | exp Substance-Related Disorders/ | 283084 |
| 10 | exp Illicit Drugs/ | 12765 |
| 11 | (substance* adj3 "use").mp. | 43225 |
| 12 | (substance* adj3 abuse).mp. | 55275 |
| 13 | (abusing adj3 substance*).mp. | 1130 |
| 14 | (drug adj3 abus*).mp. | 25002 |
| 15 | "drug use".mp. | 47430 |
| 16 | (substance* adj3 disorder*).mp. | 107604 |
| 17 | (substance* adj3 misus*).mp. | 3331 |
| 18 | (drug? adj3 misus*).mp. | 4903 |
| 19 | (drug? adj3 ad?ict*).mp. | 15482 |
| 20 | (substance? adj3 ad?ict*).mp. | 2459 |
| 21 | (illicit adj3 drug?).mp. | 20669 |
| 22 | (substance* adj3 depend*).mp. | 5326 |
| 23 | (drug? adj3 depend*).mp. | 17131 |
| 24 | exp Alcohol Drinking/ | 70483 |
| 25 | Drug-Seeking Behavior/ | 1390 |
| 26 | Driving Under the Influence/ | 657 |
| 27 | exp "Marijuana Use"/ | 5984 |
| 28 | exp smoking/ | 149983 |
| 29 | exp "Tobacco Use"/ | 5564 |
| 30 | exp Cocaine/ | 25585 |
| 31 | exp Alcoholic Beverages/ | 20612 |
| 32 | intoxicat*.mp. | 58066 |
| 33 | alcohol*.mp. | 436319 |
| 34 | opioid*.mp. | 126110 |
| 35 | (marijuana* or marihuana* or cannab*).mp. | 54502 |
| 36 | (heroin or cocaine).mp. | 59604 |
| 37 | (nic?otine* or cigarette* or cigar* or tobacco* or vape? or vaping or chaw or dipping).mp. | 208616 |
| 38 | (amphetamin* or speed).mp. | 211357 |
| 39 | (upper? or downer?).mp. | 356289 |
| 40 | 9 or 10 or 11 or 12 or 13 or 14 or 15 or 16 or 17 or 18 or 19 or 20 or 21 or 22 or 23 or 24 or 25 or 26 or 27 or 28 or 29 or 30 or 31 or 32 or 33 or 34 or 35 or 36 or 37 or 38 or 39 | 1606811 |
| 41 | 8 and 40 | 4740 |
| 42 | limit 41 to (english language and humans and "all adult (19 plus years)") | 1962 |
| 43 | limit 42 to "therapy (maximizes sensitivity)" | 500 |

APA PsycInfo

| **#** | **String** | **Yield** |
| --- | --- | --- |
| 1 | exp eating disorders/ | 31708 |
| 2 | (anorex* nervosa or bulim* nervosa or binge* eat* or purg*).mp. | 23443 |
| 3 | (eating adj4 disorder*).mp. | 31826 |
| 4 | (feeding adj4 disorder*).mp. | 1026 |
| 5 | (appetite adj4 disorder*).mp. | 140 |
| 6 | OSFED.mp. | 48 |
| 7 | ARFID.mp. | 143 |
| 8 | 1 or 2 or 3 or 4 or 5 or 6 or 7 | 43395 |
| 9 | exp "substance use disorder"/ | 132117 |
| 10 | exp drug addiction/ | 19412 |
| 11 | prescription drug misuse/ | 315 |
| 12 | (substance* adj3 "use").mp. | 66106 |
| 13 | (substance* adj3 abuse).mp. | 37682 |
| 14 | (abusing adj3 substance*).mp. | 2291 |
| 15 | (drug adj3 abus*).mp. | 58093 |
| 16 | "drug use".mp. | 35159 |
| 17 | (substance* adj3 disorder*).mp. | 23633 |
| 18 | (substance* adj3 misus*).mp. | 3871 |
| 19 | (drug? adj3 misus*).mp. | 2449 |
| 20 | (drug? adj3 ad?ict*).mp. | 18278 |
| 21 | (substance? adj3 ad?ict*).mp. | 2725 |
| 22 | (illicit adj3 drug?).mp. | 8122 |
| 23 | (substance* adj3 depend*).mp. | 5367 |
| 24 | (drug? adj3 depend*).mp. | 18033 |
| 25 | alcohol drinking patterns/ | 23594 |
| 26 | drug seeking/ | 1031 |
| 27 | driving under the influence/ | 2094 |
| 28 | marijuana usage/ | 3012 |
| 29 | nicotine/ or exp tobacco smoking/ or "tobacco use disorder"/ | 39967 |
| 30 | drug usage/ | 19260 |
| 31 | heroin addiction/ or intravenous drug usage/ | 6536 |
| 32 | intoxicat*.mp. | 10879 |
| 33 | alcohol*.mp. | 147903 |
| 34 | opioid*.mp. | 27457 |
| 35 | (marijuana* or marihuana* or cannab*).mp. | 27413 |
| 36 | (heroin or cocaine).mp. | 29732 |
| 37 | (nic?otine* or cigarette* or cigar* or tobacco* or vape? or vaping or chaw or dipping).mp. | 57703 |
| 38 | (amphetamin* or speed).mp. | 66669 |
| 39 | (upper? or downer?).mp. | 29809 |
| 40 | 9 or 10 or 11 or 12 or 13 or 14 or 15 or 16 or 17 or 18 or 19 or 20 or 21 or 22 or 23 or 24 or 25 or 26 or 27 or 28 or 29 or 30 or 31 or 32 or 33 or 34 or 35 or 36 or 37 or 38 or 39 | 415264 |
| 41 | 8 and 40 | 5666 |
| 42 | limit 41 to (human and english language and "300 adulthood <age 18 yrs and older>") | 2304 |
| 43 | limit 42 to "therapy (maximizes sensitivity)" | 1154 |
| 44 | limit 43 to "0110 peer-reviewed journal" | 915 |

Embase

| **#** | **String** | **Yield** |
| --- | --- | --- |
| 1 | exp eating disorders/ | 53357 |
| 2 | (anorex* nervosa or bulim* nervosa or binge* eat* or purg*).mp. | 41782 |
| 3 | (eating adj4 disorder*).mp. | 40285 |
| 4 | (feeding adj4 disorder*).mp. | 6982 |
| 5 | (appetite adj4 disorder*).mp. | 4391 |
| 6 | OSFED.mp. | 50 |
| 7 | ARFID.mp. | 233 |
| 8 | 1 or 2 or 3 or 4 or 5 or 6 or 7 | 77552 |
| 9 | exp "substance use disorder"/ | 240263 |
| 10 | exp drug addiction/ | 240263 |
| 11 | prescription drug misuse/ | 945 |
| 12 | (substance* adj3 "use").mp. | 57280 |
| 13 | (substance* adj3 abuse).mp. | 72453 |
| 14 | (abusing adj3 substance*).mp. | 1340 |
| 15 | (drug adj3 abus*).mp. | 85352 |
| 16 | "drug use".mp. | 171201 |
| 17 | (substance* adj3 disorder*).mp. | 29188 |
| 18 | (substance* adj3 misus*).mp. | 4756 |
| 19 | (drug? adj3 misus*).mp. | 11547 |
| 20 | (drug? adj3 ad?ict*).mp. | 20032 |
| 21 | (substance? adj3 ad?ict*).mp. | 3490 |
| 22 | (illicit adj3 drug?).mp. | 24131 |
| 23 | (substance* adj3 depend*).mp. | 7434 |
| 24 | (drug? adj3 depend*).mp. | 81263 |
| 25 | drinking behavior/ | 49732 |
| 26 | drug seeking/ | 2 |
| 27 | driving under the influence/ | 1826 |
| 28 | exp cannabis/ | 36099 |
| 29 | nicotine/ or exp tobacco smoking/ or "tobacco use disorder"/ | 433039 |
| 30 | drug usage/ | 118026 |
| 31 | heroin addiction/ or intravenous drug usage/ | 9476 |
| 32 | intoxicat*.mp. | 241997 |
| 33 | alcohol*.mp. | 679860 |
| 34 | opioid*.mp. | 134319 |
| 35 | (marijuana* or marihuana* or cannab*).mp. | 83535 |
| 36 | (heroin or cocaine).mp. | 85015 |
| 37 | (nic?otine* or cigarette* or cigar* or tobacco* or vape? or vaping or chaw or dipping).mp. | 284464 |
| 38 | (amphetamin* or speed).mp. | 270423 |
| 39 | (upper? or downer?).mp. | 523447 |
| 40 | 9 or 10 or 11 or 12 or 13 or 14 or 15 or 16 or 17 or 18 or 19 or 20 or 21 or 22 or 23 or 24 or 25 or 26 or 27 or 28 or 29 or 30 or 31 or 32 or 33 or 34 or 35 or 36 or 37 or 38 or 39 | 2531363 |
| 41 | 8 and 40 | 11283 |
| 42 | limit 41 to (human and english language and adult <18 to 64 years>) | 4183 |
| 43 | limit 42 to "therapy (maximizes sensitivity)" | 956 |

Cinahl

| **#** | **String** | **Yield** |
| --- | --- | --- |
| 1 | (MH "Feeding and Eating Disorders of Childhood+") OR (MH "Eating Disorders+") OR (MH "Binge Eating Disorder") OR (MH "Bulimia Nervosa") OR (MH "Avoidant Restrictive Food Intake Disorder") | 18800 |
| 2 | anorex* nervosa OR bulim* nervosa OR binge* eat* OR purg* | 10088 |
| 3 | eating W4 disorder* | 15618 |
| 4 | feeding W4 disorder* | 674 |
| 5 | appetite W4 disorder* | 43 |
| 6 | OSFED | 23 |
| 7 | arfid or avoidant restrictive food intake disorder or selective eating disorder | 337 |
| 8 | S1 OR S2 OR S3 OR S4 OR S5 OR S6 OR S7 | 22996 |
| 9 | (MH "Substance Dependence") OR (MH "Substance Abuse, Intravenous") | 14985 |
| 10 | (MH "Street Drugs+") OR (MH "Intravenous Drug Users") | 7981 |
| 11 | substance* W3 use | 59854 |
| 12 | substance* W3 abuse | 51890 |
| 13 | abusing W3 substance* | 68 |
| 14 | drug W3 abus* | 8200 |
| 15 | drug users or drug abusers or drug addicts or substance abuse | 75334 |
| 16 | substance* W3 disorder* | 43167 |
| 17 | substance* W3 misus* | 2163 |
| 18 | drug* W3 misus* | 1207 |
| 19 | drug* W3 ad#ict* | 2533 |
| 20 | substanc* W3 ad#ict* | 635 |
| 21 | illicit W3 drug* | 5351 |
| 22 | substance* W3 depend* | 11648 |
| 23 | drug* W3 depend* | 2821 |
| 24 | (MH "Alcohol Drinking+") OR (MH "Drinking Behavior+") | 33346 |
| 25 | driving under the influence of alcohol OR driving under the influence of marijuana | 398 |
| 26 | (MH "Cannabis+") | 9786 |
| 27 | marijuana use or cannabis use or marijuana or cannabis or pot | 19911 |
| 28 | (MH "Smoking+") | 73262 |
| 29 | (MH "Cocaine+") | 5055 |
| 30 | intoxicat* OR alcohol* OR opioid* OR ( (marijuana* or marihuana* or cannab*) ) | 164474 |
| 31 | (heroin or cocaine) | 14412 |
| 32 | (nic#otine* or cigarette* or cigar* or tobacco* or vape* or vaping or chaw or dipping | 56557 |
| 33 | amphetamin* or speed | 37248 |
| 34 | upper* or downer* | 66917 |
| 35 | S9 OR S10 OR S11 OR S12 OR S13 OR S14 OR S15 OR S16 OR S17 OR S18 OR S19 OR S20 OR S21 OR S22 OR S23 OR S24 OR S25 OR S26 OR S27 OR S28 OR S29 OR S30 OR S31 OR S32 OR S33 OR S34 | 422273 |
| 36 | S35 AND S8 | 2081 |
| 37 | Limiters - English Language; Clinical Queries: Therapy - High Sensitivity; Human; Age Groups: All Adult | 307 |

**Supplementary Table 1:** Risk of Bias Assessment

| **Study (First Author, year)** | **1.Aims** | **2.Measures** | **3.Characteristics** | **5.Confonders** | **6.Findings** | **7.Random variability** | **10.Probabilities** | **11.Representative sample** | **12.Representative accepted** | **13.Standard facilities** | **16.Data dredging** | **18.Statistics** | **20.Accurate measures** | **25.Confound adjustment** | **TOTAL** |
| --- | --- | --- | --- | --- | --- | --- | --- | --- | --- | --- | --- | --- | --- | --- | --- |
| Anzengruber 2006 | 1 | 1 | 1 | 0 | 1 | 1 | 1 | 1 | 1 | 1 | 1 | 1 | 1 | 0 | 12 |
| Blinder 2006 | 1 | 1 | 1 | 2 | 1 | 1 | 1 | U | U | 1 | 1 | 1 | 1 | 1 | 13 |
| Bodell 2013 | 1 | 1 | 1 | 1 | 1 | 1 | 1 | 1 | U | 1 | 1 | 1 | 1 | 1 | 13 |
| Braun 1994 | 1 | 1 | 0 | 0 | 1 | 0 | 0 | 1 | U | 1 | 1 | 1 | 1 | 0 | 8 |
| Bulik 1991 | 1 | 1 | 1 | 2 | 1 | 1 | 1 | 1 | U | 1 | 1 | 1 | 1 | 1 | 14 |
| Bulik 2008 | 1 | 1 | 1 | 1 | 1 | 1 | 1 | 1 | 1 | 1 | 1 | 1 | 1 | 1 | 14 |
| Burgalassi 2013 | 1 | 1 | 1 | 0 | 1 | 0 | 0 | U | 0 | 1 | 1 | 1 | 1 | 0 | 8 |
| Carlat 1997 | 1 | 1 | 1 | 0 | 1 | 1 | 1 | 1 | 1 | 1 | 1 | 1 | 1 | 0 | 12 |
| Casper 1996 | 1 | 1 | 1 | 1 | 1 | 1 | 0 | 1 | U | 1 | 1 | 1 | 1 | 0 | 11 |
| Corbridge 1996 | 1 | 1 | 0 | 1 | 1 | 0 | 0 | 1 | 1 | 1 | 1 | 1 | 1 | 1 | 11 |
| Corcos 2001 | 1 | 1 | 0 | 0 | 1 | 0 | 0 | U | U | U | 1 | 1 | 1 | 0 | 6 |
| Deter 1994 | 1 | 1 | 1 | 0 | 1 | 1 | 1 | 0 | U | 1 | 1 | 1 | 1 | 0 | 10 |
| Eddy et al. 2002 | 1 | 1 | 1 | 1 | 1 | 0 | 1 | 1 | 1 | 1 | 1 | 1 | 1 | 0 | 12 |
| Fairburn 1999 | 1 | 1 | 1 | 2 | 1 | 1 | 1 | 1 | 1 | 1 | 1 | 1 | 1 | 1 | 15 |
| Fichter 1999 | 1 | 1 | 1 | 0 | 1 | 1 | 0 | 1 | 1 | 1 | 1 | 1 | 1 | 0 | 11 |
| Fichter 2006 | 1 | 1 | 1 | 0 | 1 | 1 | 0 | 1 | 1 | 1 | 1 | 1 | 1 | 0 | 11 |
| Fioravanti 2014 | 1 | 1 | 1 | 0 | 1 | 1 | 1 | 1 | U | 1 | 1 | 1 | 1 | 0 | 11 |
| Franko 2005 | 1 | 1 | 1 | 0 | 1 | 1 | 1 | 1 | U | 1 | 1 | 1 | 1 | 0 | 11 |
| Franko 2008 | 1 | 1 | 1 | 1 | 1 | 1 | 1 | 1 | U | 1 | 1 | 1 | 1 | 0 | 12 |
| George 2005 | 1 | 1 | 1 | 0 | 1 | 1 | 1 | U | 0 | U | 1 | 1 | 1 | 0 | 9 |
| Hall 1984 | 1 | 1 | 1 | 0 | 1 | 1 | 0 | U | U | 1 | 1 | 1 | 1 | 0 | 9 |
| Haug 2001 | 1 | 1 | 1 | 0 | 1 | 1 | 0 | U | 0 | 0 | 1 | 1 | 1 | 0 | 8 |
| Henzel 1984 | 1 | 1 | 1 | 0 | 1 | 0 | 0 | 0 | 0 | 1 | 1 | 0 | 1 | 0 | 7 |
| Herzog 1991 | 1 | 1 | 1 | 1 | 1 | 1 | 1 | 1 | U | 1 | 1 | 1 | 1 | 1 | 13 |
| Herzog 1999 | 1 | 1 | 1 | 0 | 1 | 1 | 1 | 1 | 1 | 1 | 1 | 1 | 1 | 0 | 12 |
| Herzog 2005 | 1 | 1 | 1 | 0 | 1 | 0 | 1 | 1 | U | 1 | 1 | 1 | 1 | 0 | 10 |
| Hudson 1983 | 1 | 1 | 1 | 0 | 1 | 0 | 0 | 1 | 1 | 1 | 1 | 1 | 1 | 0 | 10 |
| Iwasaki 2000 | 1 | 1 | 1 | 1 | 1 | 1 | 0 | 1 | 1 | 1 | 1 | 1 | 1 | 0 | 12 |
| Jordan 2003 | 1 | 1 | 1 | 0 | 1 | 1 | 0 | 1 | 1 | 1 | 1 | 1 | 1 | 0 | 11 |
| Jordan 2008 | 1 | 1 | 1 | 1 | 1 | 0 | 1 | 1 | U | 1 | 1 | 1 | 1 | 0 | 11 |
| Kask et al. 2016 | 1 | 1 | 1 | 1 | 1 | 1 | 1 | 1 | 1 | 1 | 1 | 1 | 1 | 1 | 14 |
| Kask et al. 2017 | 1 | 1 | 1 | 2 | 1 | 1 | 1 | 1 | 1 | 1 | 1 | 1 | 1 | 1 | 15 |
| Kirkpartrick 2018 | 1 | 1 | 1 | 0 | 1 | 1 | 1 | 1 | 1 | 1 | 1 | 1 | 1 | 0 | 12 |
| Krahn 1991 | 1 | 1 | 1 | 0 | 1 | 1 | 0 | 1 | U | 1 | 1 | 1 | 1 | 0 | 10 |
| Krug 2008 | 1 | 1 | 1 | 0 | 1 | 1 | 1 | 1 | 1 | 1 | 1 | 1 | 1 | 0 | 12 |
| Laessle 1989 | 1 | 1 | 1 | 0 | 1 | 1 | 0 | U | U | 1 | 1 | 1 | 1 | 0 | 9 |
| Machado 2004 | 1 | 1 | 1 | 0 | 1 | 1 | 0 | U | U | 0 | 1 | 1 | 1 | 0 | 8 |
| Mann 2014 | 1 | 1 | 1 | 0 | 1 | 1 | 1 | 1 | 1 | 1 | 1 | 1 | 1 | 0 | 12 |
| Milos 2003 | 1 | 1 | 1 | 0 | 1 | 1 | 0 | 1 | 1 | 1 | 1 | 1 | 1 | 0 | 11 |
| Nagata 2002 | 1 | 1 | 1 | 2 | 1 | 1 | 1 | 1 | U | 1 | 1 | 1 | 1 | 1 | 14 |
| Nagata et al. 2000 | 1 | 1 | 1 | 0 | 1 | 1 | 1 | U | U | U | 1 | 1 | 1 | 0 | 9 |
| Nagata et al. 2003 | 1 | 1 | 1 | 0 | 1 | 1 | 1 | U | U | U | 1 | 1 | 1 | 0 | 9 |
| Nozoe 1995 | 1 | 1 | 1 | 0 | 1 | 0 | 0 | 1 | 1 | 1 | 1 | 1 | 1 | 0 | 10 |
| Selby 1995 | 1 | 1 | 1 | 0 | 1 | 0 | 0 | U | U | U | 1 | 1 | 1 | 0 | 7 |
| Strober 1996 | 1 | 1 | 1 | 2 | 1 | 1 | 1 | U | U | 1 | 1 | 1 | 1 | 1 | 13 |
| Sullivan 1998 | 1 | 1 | 1 | 1 | 1 | 1 | 0 | 1 | 1 | 1 | 1 | 1 | 1 | 0 | 12 |
| Tanaka 2001 | 1 | 1 | 1 | 0 | 1 | 1 | 1 | 1 | 1 | 1 | 1 | 1 | 1 | 0 | 12 |
| Toner 1986 | 1 | 1 | 1 | 0 | 1 | 0 | 0 | U | U | U | 1 | 1 | 1 | 0 | 7 |
| Ulfvebrand 2015 | 1 | 1 | 1 | 0 | 1 | 1 | 1 | 1 | 1 | 1 | 1 | 1 | 1 | 0 | 12 |
| Wiederman 1995 | 1 | 1 | 1 | 1 | 1 | 1 | 0 | U | 0 | U | 1 | 1 | 1 | 1 | 10 |
| Wiederman 1996 | 1 | 1 | 1 | 0 | 1 | 1 | 0 | 1 | 0 | 1 | 1 | 1 | 1 | 0 | 10 |
| Wiseman 1998 | 1 | 1 | 1 | 0 | 1 | 1 | 1 | U | 0 | 1 | 1 | 1 | 1 | 0 | 10 |
| **Total** | 52 | 52 | 49 | 24 | 52 | 40 | 29 | 35 | 22 | 43 | 52 | 51 | 52 | 12 | 10.87 |

## **Supplementary Table 2.** Outcomes related to AN and SUDs or Substance Use (N=52)

| **Author** | **Year** | **Eating Disorder and SUDS Results** |
| --- | --- | --- |
|  |  |  |
| Anzengruber et al. | 2006 | - Women with EDs reported higher smoking rates and nicotine dependence than controls - Among women with EDs, highest rates of smoking were present in those with binge/purge subtypes - Smoking was associated with impulsive personality traits in those with EDs - Although both groups began smoking at ~16 years old, smoking tended to occur after ED onset among the clinical group |
| Blinder et al. | 2006 | - 22% had comorbid SUD, with significant differences across EDs - SUDs were half as likely to co-occur with AN-R compared to the other EDs |
| Bodell et al. | 2013 | - Lifetime comorbidity of SUD with AN was 16.7% |
| Braun et al. | 1994 | - Among those with AN-R, lifetime frequency of: alcohol dependence was 5.9%, any alcohol or drug dependence was 11.8% - Patients with AN-R were significantly less likely to be alcohol dependent or any drug dependent compared to the sample as a whole |
| Bulik et al. | 1992 | - Rates of substance use among those with AN: 5.8% cigarettes, 11.1% alcohol, 74.1% caffeine, 7.7% amphetamine, 3.8% barbiturate, 3.8% cocaine, 3.8% diazepam, 15.4% marijuana, 3.8% quaaludes - Women with AN were significantly less likely than those with BN to use cigarettes, alcohol, laxatives, marijuana, and amphetamines |
| Bulik et al. | 2008 | - Of the 412 participants that had available data, 25.7% (n=106) reported psychoactive substance abuse and dependence |
| Burgalassi et al. | 2009 | - Caffeine abuse was significantly higher among ED patients than controls - There was no significant difference between ED groups in dependence, intoxication, or withdrawal |
| Carlat et al. | 1997 | - Among those with AN, 17% (n=5) reported substance abuse (primarily alcohol or cocaine) - 14% (n=4) of AN patients had a family history of alcoholism |
| Casper et al. | 1996 | - At 8 year follow-up, AN patients were classified as having a good, intermediate, or poor outcomes - 17% of “good” and 7.7% of “intermediate” groups reported alcohol abuse; whereas, 2.1% of “good” group reported alcohol dependence - Tobacco dependence was present in 10.6% of “good” and 15.4% of “intermediate” groups - Drug abuse was present in 7.7% of “intermediate” and 11.1% of “poor” groups; no drug dependence was reported |
| Corbridge & Bell | 1996 | - 0% of patients with AN reported alcohol or drug misuse - Low comorbidity may suggest substance use was underreported in sample |
| Corcos et al. | 2001 | - Those with AN-R showed significantly less drug use and alcohol abuse and/or dependence than AN-P and BN participants - More than half of ED patients regularly consume psychotropics; no difference in consumption between diagnoses |
| Deter & Herzog | 1994 | - At follow-up, 13.5% of patients reported substance abuse; 0% of which met Morgan/Russell criteria for “good” outcomes, 27% “good” but disturbed eating, 14% “intermediate”, and 44% “poor” |
| Eddy et al. | 2002 | - History of drug abuse was significantly higher among those with AN-BP (13%) compared to AN-R with no history of B/P (0%); remained after controlling for illness duration - History of alcohol [drug] abuse was 4%, 11%, and 19% [0%, 13%, 16%] among those with AN-R with no history of B/P, AN-R with history of B/P, and AN-BP, respectively |
| Fairburn et al. | 1999 | - Among those with AN, 6% (n=4) reported premorbid drug abuse, and 13% (n=7) reported premorbid alcohol abuse |
| Fichter & Quadflieg | 1999 | - For the whole AN sample, SUD comorbidity at 6 year follow-up was 20.0%, and lifetime comorbidity obtained at both follow-ups for SUD was 39.8% |
| Fichter et al. | 2006 | - Among those with AN at 12 year follow-up, lifetime and current prevalence of SUD were 29.9% and 9.1%, respectively - Drug dependence (mostly prescription drugs) accounted for the majority of SUDs, with 16.9% lifetime and 2.6% current drug dependence at 12 year follow-up |
| Fioravanti et al. | 2014 | - 3.9% of patients with AN-R and AN-BP fulfilled criteria for lifetime diagnosis of cocaine or amphetamine abuse |
| Franko et al. | 2005 | - Lifetime history of AUD was present in 24% (n=33) of patients with AN - History or occurrence of AUD did not influence recovery time - Among AN patients, onset of AUD was predicted by: poor psychosocial functioning, history of substance use, depression, body image concerns, and vomiting - AUD recovery among those with AN was predicted by group therapy and hospitalization |
| Franko et al. | 2008 | - The prevalence of DUD (history or prospective onset during study) among those with AN was 16% (n=22) - History or occurrence of DUD did not influence recovery time - For AN, significant predictors for the onset of DUD were: alcohol use, suicide attempts, and hospitalization prior to study for an affective disorder |
| George & Waller | 2005 | - Smoking was less prevalent in AN compared to BN and the control group (mood disorders) - Among those with AN, 24% (n=6) were current smokers and 44% (n=11) had ever smoked - Women with EDs had higher motivation to smoke, both generally and for weight control, than the control group - Coping with stress was the strongest smoking motivator - There were similar dependence levels between groups |
| Hall et al. | 1984 | - At follow-up, two AN patients (5%) met criteria for alcohol abuse disorder; both reported long-standing binge vomiting and were categorized as having “intermediate” outcomes |
| Haug et al. | 2001 | - Among those with AN, 37% reported smoking, 85.9% [33.8%] caffeine use [abuse], 35.4% [7.8%] alcohol use [abuse], 12.3% [4.7%] marijuana use [abuse] - Those with AN were significantly less likely to smoke occasionally and use alcohol compared to those with BN - In comparison to those with AN-R, purgers (AN-P and BN) were significantly more likely to exhibit alcohol abuse and consume higher amounts of caffeine |
| Henzel | 1984 | - 33% (n=5) of the sample scored in the alcoholic range, with an additional 27% scoring “uncertain” - Based on the Brief MAST score, 53% of participants would be diagnosed as alcoholic |
| Herzog et al. | 1999 | - 5.9% of those with AN-R and 16.1% of those with AN-BP had a lifetime history of SUD |
| Herzog et al. | 1992 | - 0 AN patients and 8% of ANBN patients had substance abuse diagnoses - Lifetime prevalence of SUD was lower among those with AN (12%) than BN (31%) and ANBN (37%) but this did not reach statistical significance |
| Herzog et al. | 2006 | - Lifetime history of DUD was 16% (n=22) among those with AN, 5 with AN-R and 17 with AN-BP - The most commonly abused illicit drugs were amphetamines, cocaine, and marijuana - There was no differences in DUD between diagnostic groups; however, there was a trend toward amphetamine abuse being more likely among those with AN |
| Hudson et al. | 1983 | - 6% (n=1) of AN patients, and 36% (n=9) of ANBN patients had comorbid alcohol abuse or dependence   13% (n=2) of AN patients, and 28% (n=7) of ANBN patients had comorbid amphetamine abuse or dependence   - 19% (n=3) of AN patients, and 24%(n=6) of ANBN patients had other comorbid substance abuse - In total, 19% (n=3) of AN patients, and 44% (n=11) of ANBN patients had at least one diagnosis of a substance use disorder |
| Iwasaki et al. | 2000 | - Lifetime prevalence of alcohol abuse was 3% (n=2) in those with AN-R, and 14% (n=5) in those with AN-BP - Lifetime prevalence of both sedative and inhalant abuse was 3% (n=3) among those with AN-BP, no AN-R patient was diagnosed with either sedative or inhalant abuse - In total, 3% (n=2) of AN-R patients, and 14% (n=5) of AN-BP patients met criteria for lifetime substance use disorder |
| Jordan et al. | 2003 | - Among those with AN, 28% (n=11) met criteria for alcohol abuse/dependence, 20% (n=8) for cannabis abuse/dependence, and 33% (n=13) for lifetime psychoactive SUD - Prevalence of psychoactive SUD was similar between AN subtypes, and between the AN and control (depression) groups |
| Jordan et al. | 2008 | - Among AN patients, 34% met criteria for any psychoactive SUD, 27% for alcohol abuse/dependence, 21% for cannabis abuse/dependence, and 9% for other psychoactive SUD - The AN and depression groups had lower levels of any psychoactive SUD and alcohol abuse/dependence compared to those with BN - There were significant differences in SUD prevalence between AN subtypes; however, there was a trend toward higher prevalence of any psychoactive SUD and cannabis abuse/dependence among those with AN-BP compared to AN-R |
| Kask et al. | 2016 | - Mortality from natural causes in patients with AN was 10x higher among those with comorbid alcohol use disorder and other substance disorder compared to the general female population - Risk of all-cause mortality was twice as high in patients with AN and comorbid AUD or other SUD compared to controls with the same disorder - AUD is associated with high rates of mortality in AN for both unnatural and natural causes of death |
| Kask et al. | 2017 | - Those with AN and comorbid alcohol or other SUD had nearly 20- and 30-fold increases in mortality, respectively - Among the 49 patients with comorbid AUD, 19 (39%) died during follow-up |
| Kirkpatrick et al. | 2019 | - Among the 73 adolescents with AN, 42.5% (n=31) reported substance use - ED severity did not differ between substance users and non-users before or after treatment; however, substance users were more likely to drop out of treatment (41.5% vs. 25.2%) |
| Krahn et al. | 1991 | - Only 7.1% (n=1) of the AN sample was classified as having high caffeine use (>750mg/day), compared to 18.7% (n=17) of the BN sample - Differences between diagnoses in high caffeine use were not significant |
| Krug et al. | 2008 | - For most lifetime and current tobacco, drug use, and comorbid substance use variables, those with AN-BP had the highest prevalence, and those with AN-R had the lowest - AN-R patients had higher lifetime and current alcohol use than BN but not AN-BP patients - AN-R patients are comparable to controls in terms of risk for abuse of all kinds of substances |
| Laessle et al. | 1989 | - AN-R patients had a significantly lower lifetime frequency of total substance use disorders when compared to bulimic patients (AN-BP, BN, BN with history of AN) - 4.8% (n=1) of AN-R patients had drug/medication abuse/dependence, and 20% (n=4) of AN-BP patients had alcohol abuse/dependence |
| Machado et al. | 2004 | - Among patients with AN, 7.9% (n=5) reported alcohol abuse, and 0 reported drug abuse |
| Mann et al. | 2014 | - Among adolescents with AN, lifetime prevalence of any substance use was 24.6% - Alcohol, cannabis, and tobacco were most frequently used; cannabis was the most commonly abused substance |
| Milos et al. | 2003 | - Lifetime diagnosis of a substance-related disorder was found in 22% (n=17) of those with AN - No significant difference in prevalence of substance-related disorder between AN and BN |
| Nagata et al. | 2000 | - 10% (n=6) of patients with AN-R and 21% (n=13) of patients with AN-BP met criteria for habitual drinking (regular drinking ≥4 times/week) - 3% (n=2) of those with AN-R and 15% (n=9) of those with AN-BP were heavy regular alcohol drinkers (drunk at least 1/per week OR ≥4 12-oz beers/equivalent per day at least 4 days/week) - 2% (n=1) of those in the AN-R and AN-BP groups engaged in substance use |
| Nagata et al. | 2002 | - 3.2% (n=2) of AN-R patients and 12.5% (n=6) of AN-BP patients had lifetime diagnosis of DUD (predominantly solvent fumes or benzodiazepines) - DUD was associated with parental loss during childhood, borderline personality disorder, and history of conduct disorder - There were no significant differences in ED symptoms between those with and without DUD |
| Nagata et al. | 2003 | - 3% (n=2) of patients with AN-R and 17% (n=8) of patients with AN-BP had lifetime history of DUD - Among the ED+DUD group, there was dependency (4 methamphetamine, 6 inhalants, 6 tranquilizers) and abuse (5 inhalants, 2 tranquilizers) |
| Nozoe et al. | 1995 | - AN patients with a history of stimulant (alcohol, coffee, and/or cigarettes) abuse after illness onset required a longer period of in-patient treatment |
| Selby et al. | 1995 | - Among those with AN, 20% (n=5) reported a personal substance misuse problem |
| Strober et al. | 1995 | - 18.9% (n=18) patients developed SUD during the 10 year follow-up period; 11 met abuse criteria and 7 met dependence criteria - Compared to restrictors, those who were engaging in binge eating at admission had an increased risk (5.8x) of SUD and greater likelihood of having at least one first-degree relative with SUD |
| Sullivan et al. | 1998 | - Alcohol dependence was significantly more common in the AN group compared to the control group - Among AN patients at 12 year follow-up, 30% (n=21) met criteria for lifetime diagnosis of any drug dependence, 27.1% (n=19) for alcohol dependence, 8.6% (n=6) for cannabis dependence, and 2.9% (n=2) for other drug dependence |
| Tanaka et al. | 2001 | - 8.2% (n=5) of AN patients met criteria for alcohol abuse at referral - At follow up, 57% (n=4) of the deceased group and 7% (n=1) of the poor outcome group met criteria for alcohol abuse compared to 0% of the good and intermediate outcome groups; these differences were statistically significant |
| Toner et al. | 1986 | - Those with AN-B had a higher incidence of SUDs (42.9%) during the last year compared to those with AN-R (0%) and the comparison group (7.7%) - Tobacco use disorder (lifetime prevalence and last year incidence) was higher among those with AN-B than AN-R |
| Ulfvebrand et al. | 2015 | - 2.8% (n=25) of ANR patients had comorbid alcohol dependence, and 0.6% (n=5) had comorbid alcohol abuse - 1.2% (n=11%) of ANR patients had comorbid substance dependence, and 0.3% (n=3) had comorbid substance abuse - In total, 4.2% (n=37) of ANR patients had any comorbid substance use disorder - 7% (n=32) of ANBP patients had comorbid alcohol dependence, and 1.5% (n=7) of ANBP had comorbid alcohol abuse - 4% (n=18) of ANBP patients had comorbid substance dependence, and 1.8% (n=8) of ANBP had comorbid substance abuse |
| Wiederman & Pryor | 1996 A | - Women with AN were less likely than those with BN to have used alcohol, amphetamines, barbiturates, marijuana, tranquilizers, and cocaine, even when controlling for age and symptom severity - Irrespective of diagnosis, severity of: restriction was predictive of amphetamine use; binge eating was predictive of tranquilizer use; purging was predictive of alcohol, cocaine, and cigarette use |
| Wiederman & Pryor | 1996 B | - Those with AN-BP (n=13) constituted a minority (22%) of the AN group but accounted for the majority of the substance use - The incidence of alcohol, cigarette, and marijuana use among exclusively restricting anorexics was 10.97%, 8.7%, and 4.3%, respectively |
| Wiseman et al. | 1998 | - Those with AN-R were less likely to regularly smoke (13.64%) than nonclinical controls (38.10%) and those with AN-BP or BN (58.82%) - ED patients who smoked had higher drive for thinness, body dissatisfaction, and interceptive awareness scores than patients who did not smoke - Age predicted smoking among ED patients |

**Abbreviations:** Eating disorder (ED); substance use disorder (SUD); anorexia nervosa-restricting type (AN-R); anorexia nervosa (AN); bulimia nervosa (BN); anorexia nervosa-purging type (AN-P); anorexia nervosa, binge-eating/purging type (AN-BP); alcohol use disorder (AUD); drug use disorder (DUD); anorexia nervosa and bulimia nervosa (ANBN); anorexia nervosa, bingeing type (AN-B)

**Supplementary Figure 1.** Forest Plot of the Prevalence of Substance Use Disorders by AN-R and AN-BP

**
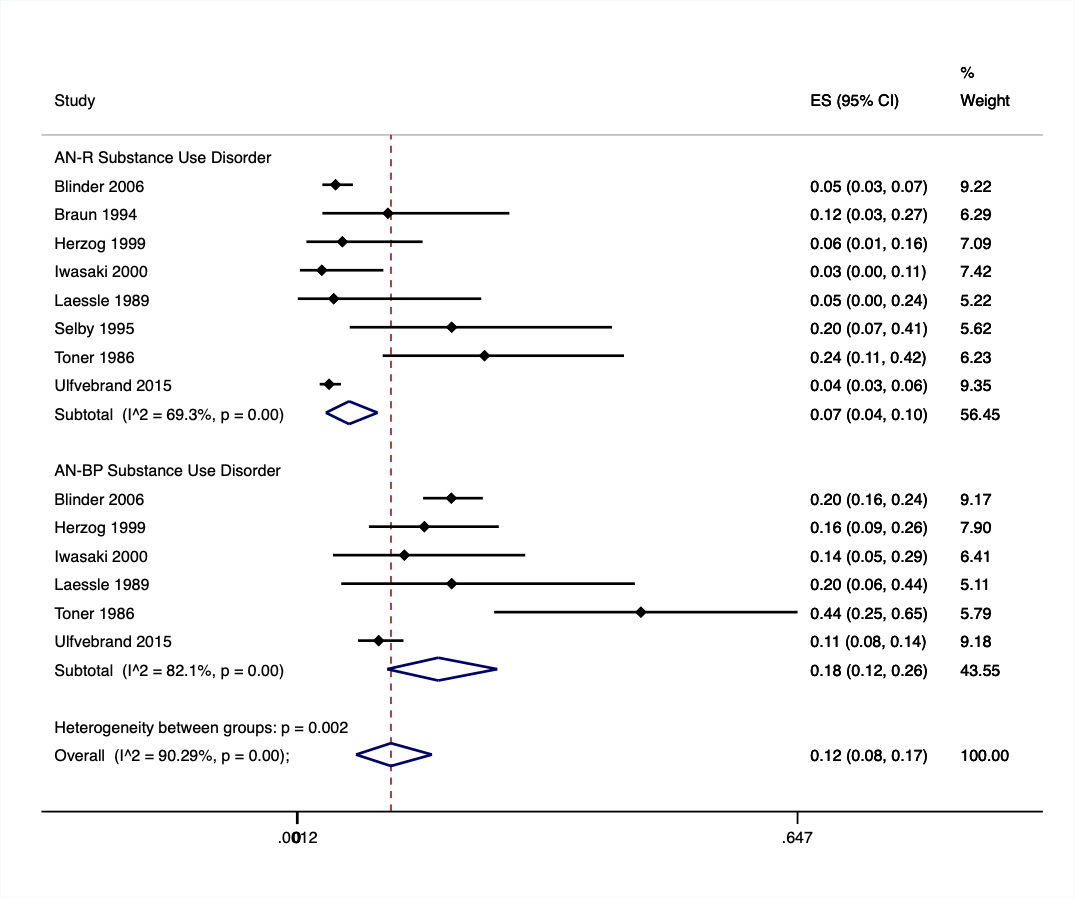
**

Notes: Blue diamond represents the pooled effects per AN type and the overall pooled effect, Red dashed line represents the overall pooled effect, ES = effect size.

**Supplementary Figure 2.** Forest Plot of the Prevalence of Drug Abuse/Dependance by AN-R and AN-BP

**
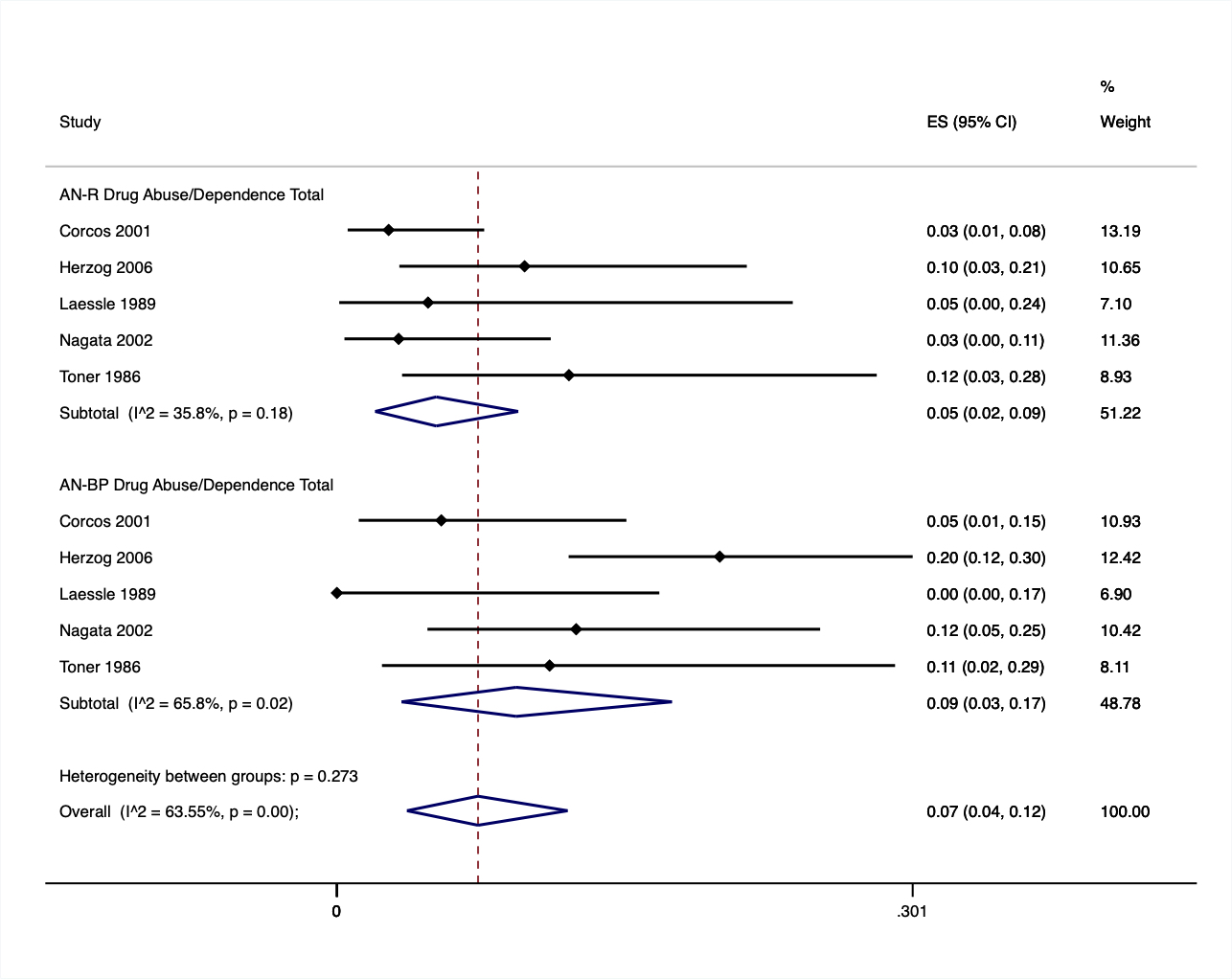
**

Notes: Blue diamond represents the pooled effects per AN type and the overall pooled effect, Red dashed line represents the overall pooled effect, ES = effect size.
